# Vorinostat and quinacrine have synergistic effects in T-cell acute lymphoblastic leukemia through reactive oxygen species increase and mitophagy inhibition

**DOI:** 10.1038/s41419-018-0679-6

**Published:** 2018-05-22

**Authors:** Bo Jing, Jin Jin, Rufang Xiang, Meng Liu, Li Yang, Yin Tong, Xinhua Xiao, Hu Lei, Wei Liu, Hanzhang Xu, Jiong Deng, Li Zhou, Yingli Wu

**Affiliations:** 1 Key Laboratory of Cell Differentiation and Apoptosis of the Chinese Ministry of Education, Chemical Biology Division of Shanghai Universities E-Institutes, Shanghai Tongren Hospital/Faculty of Basic Medicine, Hongqiao International Institute of Medicine, Shanghai Jiao Tong University School of Medicine, 200025 Shanghai, China; 2 Department of Hematology, Rui-Jin Hospital, Shanghai Jiao Tong University School of Medicine, No.197, Ruijin Er Road, Shanghai, China; 3 Shanghai First People’s Hospital, Shanghai Jiao Tong University School of Medicine, 200011 Shanghai, China

## Abstract

Despite recent progress in the treatment, the outcome of adult acute T-cell lymphoblastic leukemia (T-ALL) is poor. Development of novel approach to combat this disease is urgently required. Vorinostat, a pan-histone deacetylase (HDAC) inhibitor, exerts promising anticancer activity in a variety of solid and hematologic malignancies. However, the efficacy of vorinostat monotherapy is unsatisfactory. Here, we show that quinacrine (QC), an anti-malaria drug with potent autophagy inhibitory activity, could synergistically enhance vorinostat-induced cell death at a non-toxic concentration. Compared to the single treatment, QC plus vorinostat significantly induced apoptosis, disrupted the mitochondrial transmembrane potential, and decreased Mcl-1 and Bcl-2/Bax ratio. Interestingly, the application of QC plus vorinostat resulted in mitophagy blockade, as reflected by the increase in the K63-linked ubiquitination of mitochondria protein and the formation of mitochondrial aggresomes. QC plus vorinostat markedly increased the reactive oxygen species (ROS) level in cells. Moreover, the ROS scavenger *N*-acetylcysteine (NAC) abrogated QC plus vorinostat-induced ROS, decreased the ubiquitination of mitochondria proteins, and cell death. Finally, using a xenograft mouse model, we demonstrated that QC plus vorinostat significantly reduced cell proliferation and induced cell death in vivo. Taken together, our results showed that the combination of QC with vorinostat may represent a novel regimen for the treatment of T-cell acute lymphoblastic leukemia, which deserves clinical evaluation in the future.

## Introduction

Acute T-cell lymphoblastic leukemia (T-ALL) is an aggressive hematologic disease, accounting for about 15 and 25% of ALL in pediatric and adult patients, respectively^1^. Despite recent progress in treatment, in contrast to children who have an approximate cure rate of 90%, less than half of adults attain disease eradication, even with hematopoietic stem cell transplantation. Even for adult ALL patients who have achieved complete remission, most of them eventually relapse and few patients are rescued even given the best therapeutic regimens currently available^2^. Therefore, the development of a novel strategy to combat this life-threatening disease is urgently required.

Histone deacetylases (HDACs), which medicate the deacetylation of histone and non-histone proteins, can regulate the activities of transcriptional factors involved in both cancer initiation and progression as well as the post-translational modification of numerous key proteins, including tumor suppressor genes^3,4^. HDACs are considered a promising anticancer drug target^5^. Vorinostat, a pan-HDAC inhibitor, has been approved by Food and Drug administration (FDA) in the United States for the treatment of cutaneous T-cell lymphoma^6^. Recently, vorinostat also exerts powerful anticancer activity in a variety of solid and hematologic malignancies including T-ALL through increasing the levels of reactive oxygen species (ROS), inducing cell apoptosis, differentiation, growth arrest, and inhibiting angiogenesis^7–11^. However, due to the activation of autophagy and other survival pathways, the anticancer effects of vorinostat are often limited^7^. Therefore, combinations of vorinostat with other agents are necessary to improve the efficacy of vorinostat.

Quinacrine (QC) is extracted from the bark of the cinchona tree and has been widely used as a dye or an antibiotic and antimalarial drug. Recent studies showed that QC inhibits the growth of several types of cancer cells and is currently undergoing phase II clinical trials^12^. Reportedly, combinations of QC with other antitumor drugs have been used for the treatment of hepatocellular carcinoma^13^, cervical cancer^14^, breast cancer^15^, prostatic cancer^16^, colorectal cancer^17^, and leukemia^18,19^. The anticancer effects are associated with suppression of autophagy, inhibition of nuclear factor-κB pathway, activation of p53 pathway, blocking of the abnormal activation of cellular heat shock responses^20^, and inhibition of the synthesis of key drug resistance proteins^21^.

In this study, we demonstrate that the combination of vorinostat with QC synergistically induce cell death of T-ALL cells in vitro and in vivo. Moreover, we show that the combination of vorinostat and QC exerts its powerful beneficial effects essentially through an increase in ROS levels and suppression of mitophagy. Our results provide the basis for further clinical evaluation of this combination for the treatment of T-ALL.

## Results

### The combination of QC and vorinostat synergistically inhibits cell viability in T-ALL cell lines

**Fig. 1:**
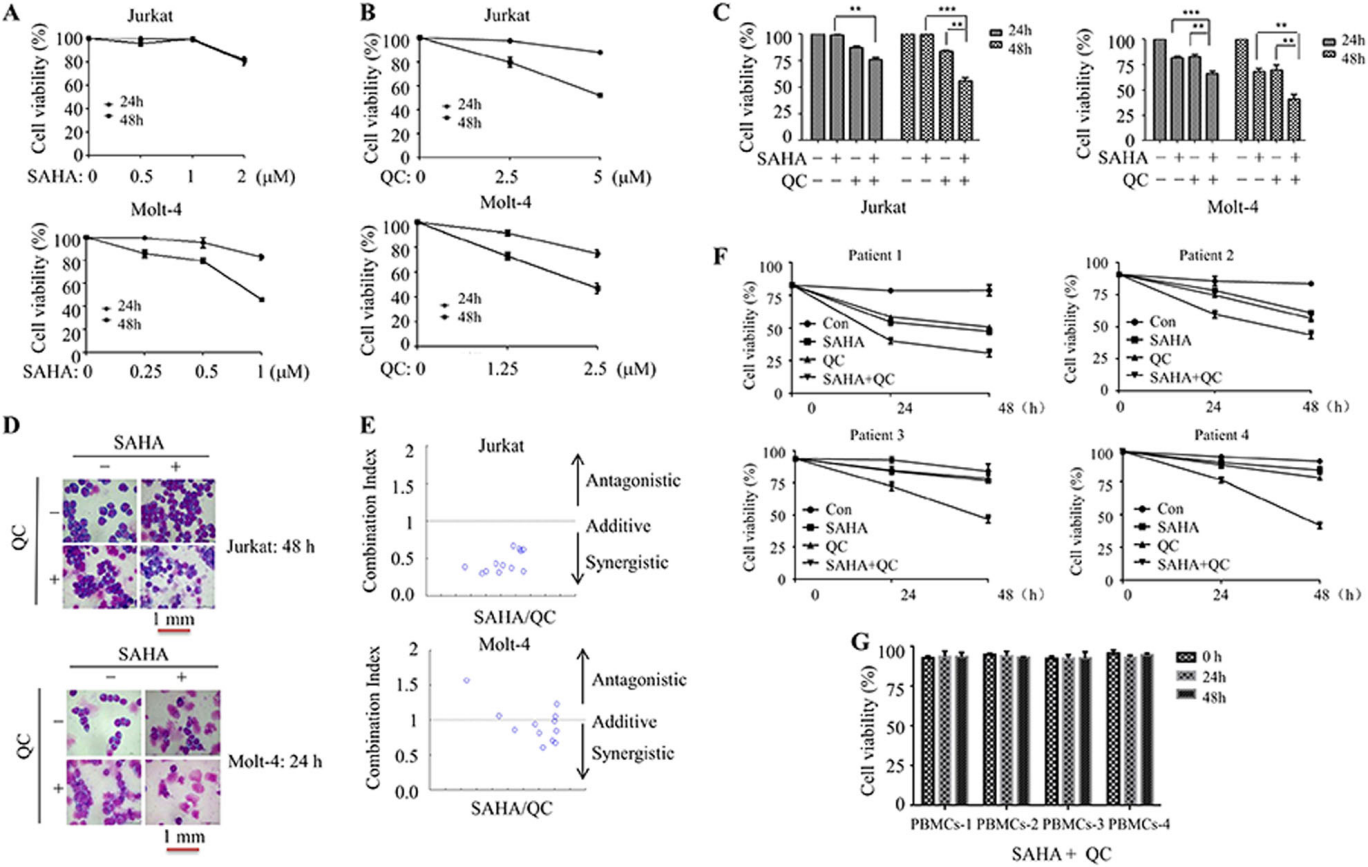
QC enhances vorinostat-inhibited cell viability in T-ALL cells. **a**, **b** Jurkat and Molt-4 cells were treated with the indicated dose of vorinostat or QC for an indicated time or the combination of both, and then the cell viability were monitored by trypan blue staining. **c**, **d** Jurkat cells were treated with vorinostat (1 μM) and/or QC (5 μM) and Molt-4 cells were treated with vorinostat (1 μM) and/or QC (2.5 μM) for an indicated time. Cell viability was monitored by trypan blue staining (**c**). The morphology of the cells was observed by Wright staining (**d**). **e** CIs were analyzed with the program CompuSyn in Jurkat and Molt-4 cells. CI value <1 indicates that the two drugs have synergic effects. **f** PBMCs from T-ALL patients were treated with vorinostat (1 μM) and QC (5 μM) for 24 and 48 h, and the cell viability was monitored by trypan blue staining. **g** PBMCs were treated with vorinostat (1 μM) and QC (5 μM) for 48 h, and the cell viability was monitored by trypan blue staining. *p < 0.05, **p < 0.01, and ***p < 0.001. All experiments were performed at least three times with the same results

Because QC was revealed to be a multifunctional drug with anticancer activity, we hypothesized that QC may enhance vorinostat-induced cell death in human T-ALL cell lines. For this purpose, Jurkat and Molt-4 cells were treated with vorinostat in the presence or absence of QC for 24 and 48 h. Compared with the single treatment (Fig. 1a–c), the combination of vorinostat and QC significantly decreased cell viability. Consistently with this finding, the morphological staining revealed that vorinostat plus QC obviously increased the disruption of cells (Fig. 1d). Next, the combination index (CI) method was used to evaluate the synergistic combinations. As can be seen in Figure 1e, Table 1, and Table 2, the combination of vorinostat with QC synergistically (CI<1.0) induced cell death. Vorinostat combined with QC also had synergistic effect in primary T-ALL cells (Fig. 1f). However, this combined effect was not observed in normal peripheral blood mononuclear cells (PBMCs) (Fig. 1g). Thus, these results suggest that vorinostat and QC have synergistic cytotoxic effects in T-ALL cell.

**Table 1:** CI values of vorinostat and QC in Jurkat cells

Dose vorinostat (μM)	Dose QC (μM)	Combination index
2	5	0.61736
2	2.5	0.32931
2	1.25	0.31165
1	5	0.63397
1	2.5	0.37588
1	1.25	0.33006
0.5	5	0.60765
0.5	2.5	0.40593
0.5	1.25	0.30434
0.25	5	0.67347
0.25	2.5	0.43356
0.25	1.25	0.38574

**Table 2:** CI values of vorinostat and QC in Molt-4 cells

Dose vorinostat (μM)	Dose QC (μM)	Combination index
2	10	1.22751
2	5	0.84567
2	2.5	0.66874
2	1.25	0.81461
1	10	1.05625
1	5	0.70538
1	2.5	0.60812
1	1.25	1.05813
0.5	10	0.97981
0.5	5	0.93742
0.5	2.5	0.85855
0.5	1.25	1.56964

### Vorinostat plus QC induces apoptosis in human T-ALL cell lines

**Fig. 2:**
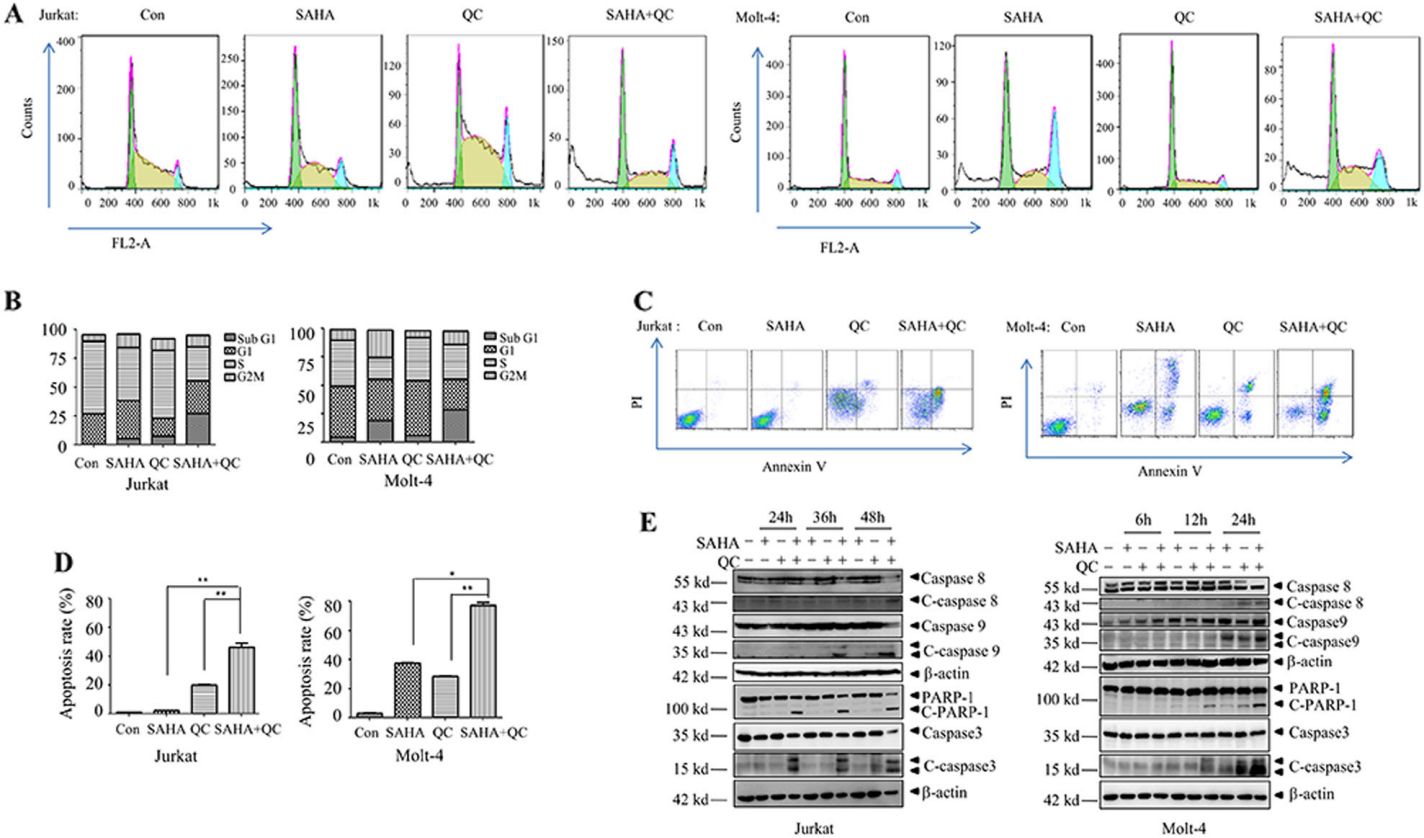
Effects of vorinostat and QC on the apoptosis of Jurkat and Molt-4 cells. Jurkat cells were treated with vorinostat (1 μM) and/or QC (5 μM) for 48 h and Molt-4 cells were treated with vorinostat (1 μM) and/or QC (2.5 μM) for 24 h. **a**, **b** Cell cycle distribution of Jurkat and Molt-4 cells was analyzed by flow cytometry. **c**, **d** Apoptosis rate of Jurkat and Molt-4 cells was established by flow cytometry analysis of Annexin-V-PI dual staining. **e** The indicated proteins were examined by western blots. *p < 0.05, **p < 0.01. All experiments were performed at least three times with the same results

We next explored the effect of vorinostat plus QC on apoptosis of T-ALL cells. As illustrated in Figure 2a, b, vorinostat plus QC significantly increased cell population in sub-G1 phase, indicating apoptotic cell death. Furthermore, we measured cell apoptosis using Annexin-V/PI staining. As can be observed in Figure 2c, d, the combination of vorinostat and QC treatment produced significantly higher numbers of apoptotic Jurkat and Molt-4 cells than the single drug treatment. Consistently with this result, vorinostat plus QC induced activation of caspase-8, caspase-9, caspase-3, and cleavage of PARP1 in Jurkat and Molt-4 cells (Fig. 2e). These data indicate that the co-treatment with vorinostat and QC significantly induces apoptosis through both extracellular and intracellular apoptosis pathways in human T-ALL cell lines.

### Vorinostat plus QC disrupts mitochondrial transmembrane potential and increases ROS in human T-ALL cells

**Fig. 3:**
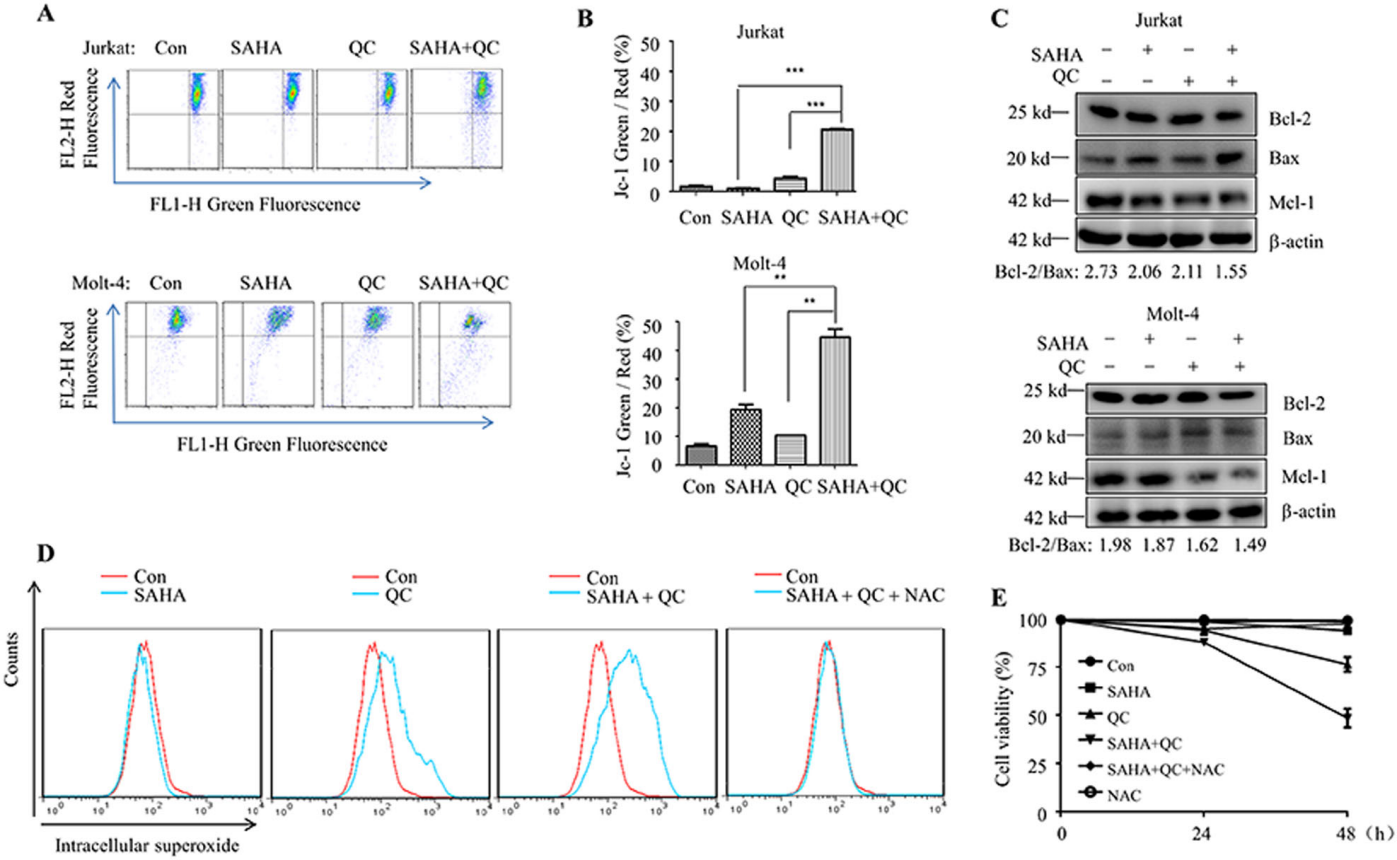
The synergic effects of vorinostat plus QC treatment on the change of mitochondrial transmembrane potential (ΔΨm). **a**–**c** Jurkat cells were treated with vorinostat (1 μM) and/or QC (5 μM) for 48 h and Molt-4 cells were treated with vorinostat (1 μM) and/or QC (2.5 μM) for 24 h. **a** ΔΨm was quantified by flow cytometry analysis of JC-1 staining. **b** Statistical analysis of **a**; **c** the indicated proteins were detected by western blotting analysis; **d** Jurkat cells were treated with vorinostat (1 μM) and/or QC (5 μM) for 30 h. DHE staining was used to analyze the level of ROS in cells; **e** Jurkat cells were treated with vorinostat (1 μM) and/or QC (5 μM) in the presence or absence of NAC (10 mM) for 48 h, and the cell viability was monitored by trypan blue staining. **p < 0.01, and ***p < 0.001. All experiments were performed at least three times with the same results

Mitochondria play a key role in apoptosis. Thus, to investigate whether mitochondrial pathways are affected by the combined effect of vorinostat and QC, we examined whether the combination disrupted the mitochondrial transmembrane potential. As expected, vorinostat plus QC caused collapse of the mitochondrial transmembrane potential (Fig. 3a, b). Because Bcl-2 family proteins have important functions in the control of the integrity of mitochondrial membrane, we assessed the expression levels of the anti-apoptotic protein Bax and the pro-apoptotic proteins Bcl-2 and Mcl-1. The co-treatment of vorinostat and QC reduced the expression of Bcl-2 and Mcl-1, and increased that of Bax, as compared to the respective levels in the single treatment group (Fig. 3c). In addition, as mitochondria are the main source of ROS, we evaluated the effect of vorinostat and/or QC on the intracellular ROS level. As shown in Figure 3d, vorinostat or QC alone increased the ROS level slightly, and their combination further augmented the level of ROS in T-ALL cells. To determine the role of ROS in the combined effect of vorinostat plus QC, we treated the cells with vorinostat plus QC in the presence of NAC, a ROS scavenger. Interestingly, NAC effectively suppressed ROS and vorinostat plus QC-induced cell death (Fig. 3e), indicating the important role of ROS in the apoptotic-inducing effect of vorinostat plus QC.

### Vorinostat plus QC blocks mitophagy and induces the formation of mito-aggresomes

**Fig. 4:**
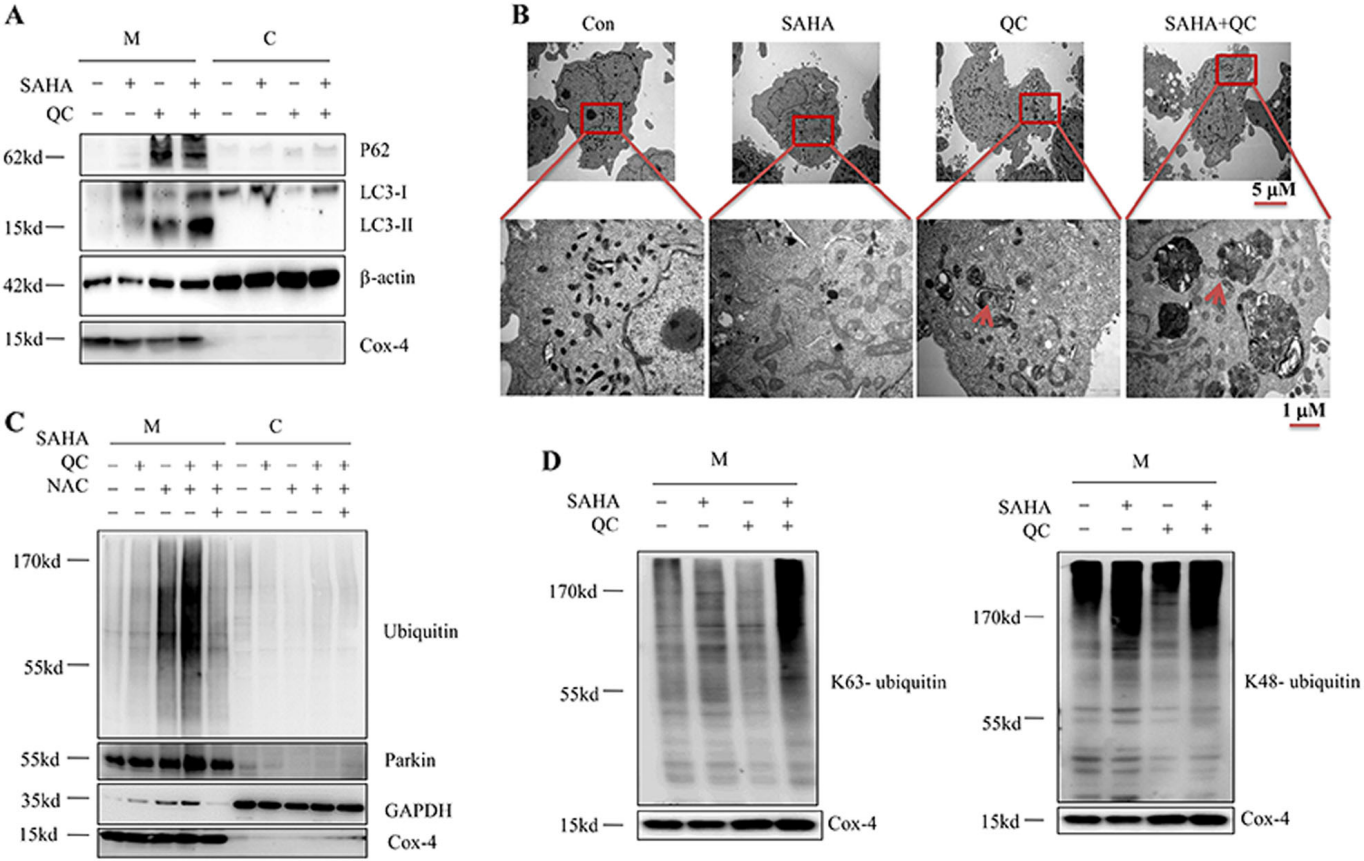
The combination of vorinostat and QC blocks mitophagy and accumulation of mito-aggresomes. (**a**, **b**, **d**) Jurkat cells were treated with vorinostat (1 μM) and/or QC (5 μM) for 36 h. **a** The indicated proteins in the cytoplasm and mitochondria were examined by western blot. **b** TEM of cells; the red arrows indicate the damaged mitochondria that are wrapping in double-membrane structures. **c** Jurkat cells treated with vorinostat (1 μM) and/or QC (5 μM) in the presence or absence of NAC (10 mM) for 36 h, and then the indicated proteins were examined by western blot. **d** The expression levels of K63-linked and K48-linked ubiquitinated proteins in the mitochondria were examined by western blot. All experiments were performed at least three times with the same results

Since the disruption of mitochondria is a crucially important aspect of the effect vorinostat plus QC exerts, and the turnover of mitochondria is regulated by mitophagy, we suspected that the examined combination may affect the mitophagy processes. The levels of the autophagy-related protein P62 and LC3-II in the mitochondria fraction were higher than those in the cytosol fraction, indicating the involvement of mitophagy changes (Fig. 4a) in the impact of the combination. Interestingly, as revealed by transmission electron microscopy (TEM), the treatment with vorinostat plus QC resulted in the accumulation of damaged mitochondria in the double-membrane autophagosome (Fig. 4b), indicating inhibition of mitophagy and formation of mito-aggresomes. Parkin, an E3 ubiquitin ligase, plays an important role in mitophagy. Once mitochondria are damaged, parkin is recruited from the cytoplasm to the mitochondrial outer membrane (MOM), where it is activated. Then, the further ubiquitination of MOM proteins results in an accumulation of mitochondrial aggregates and initial mitophagy^22^. In our study, we found that vorinostat plus QC induced a significant increase in parkin and ubiquitinated protein levels in the mitochondrial fraction but not in the cytosol fraction. Nevertheless, NAC reversed this phenomenon effectively (Fig. 4c). Furthermore, a marked increase of K63-linked ubiquitinated proteins was observed in vorinostat plus QC-treated mitochondria which are involved in mitophagyas also reported earlier^23^ (Fig. 4d). These results indicate that vorinostat plus QC blocks the flux of mitophagy and induce the formation of mito-aggresome in the cytoplasm.

### Vorinostat plus QC inhibits the proliferation of T-ALL in vivo

**Fig. 5:**
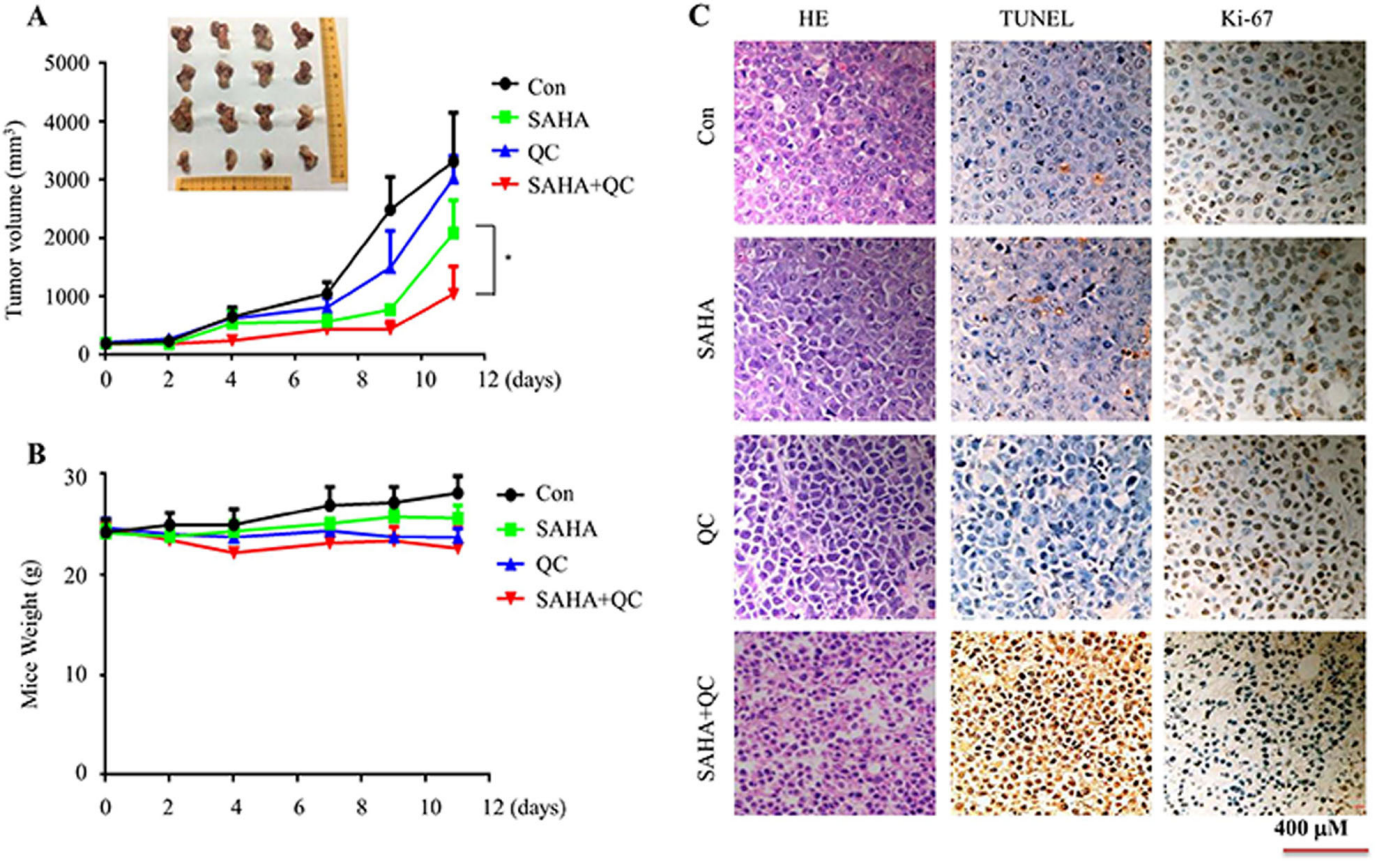
Vorinostat in combination with QC inhibits T-ALL cell growth in vivo. Mice were treated as described in material and method. **a**, **b** Tumor volume (**a**) and mice weight (**b**) were shown. **c** Mice tumor sections from each groups were subjected to immunostaining using TUNEL and anti-Ki-67 Abs. *p < 0.05

Given vorinostat plus QC effectively induces T-ALL cells death in vitro, we next investigated their combined effects in vivo using a xenograft mice model. For this purpose, Jurkat cells were injected into the right flanks of the severe combined immunodeficiency mice (SCID). When the tumor volume was palpable (approximately 100 mm^3^), mice were randomized into a treatment group and a vehicle control group (*n* = 4 per group). Mice were treated with vorinostat (70 mg/kg, intraperitoneally), QC (50 mg/kg, intraperitoneally), or with both agents daily for 12 days. The result shows that vorinostat plus QC exhibited significant antitumor activity in the Jurkat xenograft mode compared to the effects of vorinostat or QC applied alone. In addition, no overt signs of toxicity or weight loss were observed following vorinostat and/or QC treatment (Fig. 5a, b). Compared to the vehicle group or the single treatment group with vorinostat and/or QC, the combined administration of the two drugs grouped to lower tumor cell proliferation, as assessed by Ki-67 staining, and a higher percentage of apoptotic cells as evidenced by the increase of terminal deoxynucleotidyl transferase dUTP nick-end labeling-positive cells (Fig. 5c). These findings suggest that the combination of vorinostat and QC is well tolerated and efficient in inhibiting tumor growth in vivo.

## Discussion

**Fig. 6:**
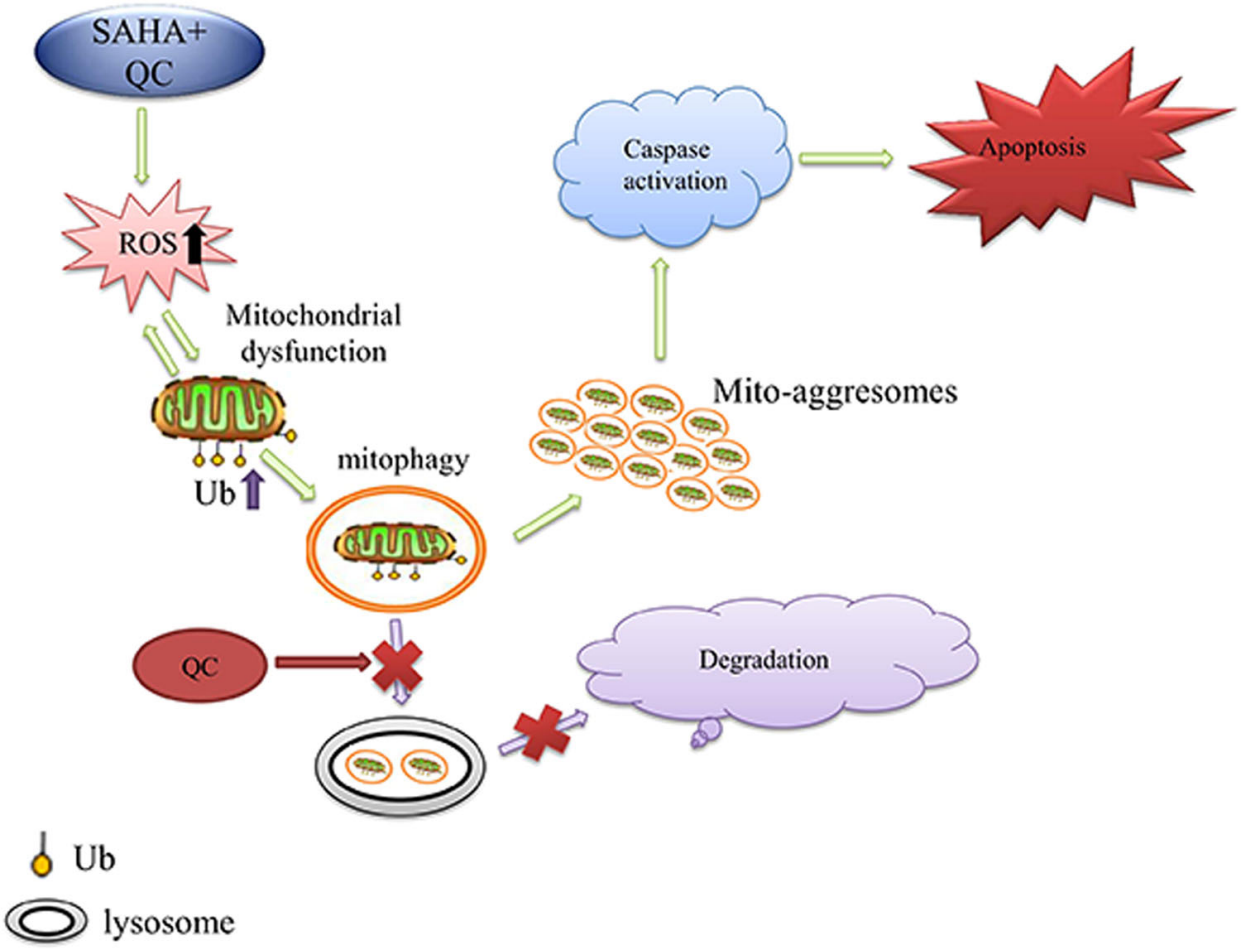
Schematic representation of the mechanisms underlying apoptosis induced by vorinostat in combination with QC. The treatment with vorinostat in combination with QC induced an increase in the level of intracellular ROS, further promoting mitochondrial dysfunction and the accumulation of K63-linked ubiquitination of the mitochondria, which indicates mitophagy. QC blocked autophagy flow through lysosomal dysfunction, resulting in the accumulation of mito-aggresomes and eventually inducing T-ALL cell death

In the present study, we found that the combination of vorinostat and QC, two clinically used drugs, could markedly inhibit T-ALL cells growth in vitro and in vivo. We showed that the increase of ROS and the inhibition of mitophagy contribute to the anti-T-ALL effect of the combination of vorinostat and QC (Fig. 6). Our results provide the basis of a novel regimen for the treatment of T-ALL.

Although vorinostat was the first HDACs inhibitor approved by the FDA for the treatment of T-cell lymphoma and has broad activity against a variety of cancers^24^, the efficacy of the single treatment with vorinostat in clinical settings is not high. A variety of combination regimens were tested in clinical studies. For example, vorinostat was used in combination with vincristine, bevacizumab, chloroquine (CQ), and others in the treatment of leukemia and metastatic clear-cell renal cell carcinoma^25–27^. One possible reason for the limited efficacy of vorinostat is that it can activate autophagy and other survival pathways in the treated cells. As expected, vorinostat induced autophagy in T-ALL cells (Supplementary Fig. S1a). Thus, we hypothesized that the inhibition of autophagy may enhance the effect of vorinostat in T-ALL cells. Recently, QC was found to be a potent autophagy inhibitor in a series of quinoline derivatives, with an effect that was approximately 60 times higher than that of CQ^28^. Moreover, QC was established to possess repositioning potential in the treatment of AML^29^. We revealed that QC markedly enhanced the effect of vorinostat in a synergistic or additive way. More importantly, we demonstrated that the combination of vorinostat and QC significantly inhibited cell proliferation and induced apoptosis in vivo. Interestingly, this combination effect could be extended to other HDAC inhibitor with QC, as panobinostat, a novel approved broad-spectrum HDAC inhibitor^30^, also has synergistic effects with QC in inducing cell death of T-ALL cells (Supplementary Fig. S2, Table 3, and Table 4). Considering the well-established safety of both drugs in clinical practice and the efficacy of their combination evidenced in the present study, it is reasonable to test this combination regimen in clinical trials.

**Table 3:** CI values of panobinostat and QC in Jurkat cells

Dose panobinostat (nM)	Dose QC (μM)	Combination index
20.0	10	0.65762
20.0	5	0.56554
20.0	2.5	0.39988
20.0	1.25	0.44391
10.0	10	0.56562
10.0	5	0.50916
10.0	2.5	0.21791
10.0	1.25	0.32257
5.0	10	0.54834
5.0	5	0.31899
5.0	2.5	0.32286
5.0	1.25	0.37940
2.5	10	0.89172
2.5	5	0.57381
2.5	2.5	0.32716
2.5	1.25	0.43103

**Table 4:** CI values of panobinostat and QC in Molt-4 cells

Dose panobinostat (nM)	Dose QC (μM)	Combination index
20.0	10	2.06834
20.0	5	0.98944
20.0	2.5	0.91221
20.0	1.25	0.88823
10.0	10	1.15378
10.0	5	0.65907
10.0	2.5	0.68349
10.0	1.25	0.64859
5.0	10	0.71968
5.0	5	0.52512
5.0	2.5	0.48863
5.0	1.25	0.57159
2.5	10	1.47339
2.5	5	0.44161
2.5	2.5	0.36865
2.5	1.25	2.05957

Mitophagy is important for the turnover of mitochondria. A series of external stimuli, including an increase of ROS, nutrition deficiency, and cell senescence, can induce mitophagy^31–33^. The damaged mitochondria are wrapped up by the autophagosome and are eventually degraded by fusion with the lysosomes, a process maintaining cell homeostasis^34^. In a previous examination, the inhibition of autophagy caused aberrant accumulation of damaged mitochondria, resulting in the release of cytochrome C, activation of apoptotic complexes, and eventually cell death^35^. We found that vorinostat plus QC markedly increased the level of the autophagy-related protein LC3-II and K63-linked ubiquitinated proteins in mitochondria, indicating an augmented level of mitophagy. However, due to the ability of QC to inhibit the fusion of lysosomes with autophagosomes, these damage mitochondria cannot be degraded by lysosome and thus accumulated in the cytosol, forming a large number of mito-aggresomes and ultimately led to apoptosis of T-ALL cells.

The role of ROS in the effect of vorinostat and QC is critical. Vorinostat and QC have been reported to increase ROS levels in a variety of cancer cells^36,37^. We found that the combination of vorinostat and QC further augmented the levels of ROS in T-ALL cells. Interestingly, the ROS scavenger NAC completely blocked the vorinostat plus QC-induced increase of ROS, the ubiquitination of mitochondrial proteins, and cell death. These findings imply that ROS are important for exerting the combined effect of vorinostat and QC. Of note, vorinostat-induced and QC-induced ROS/mitophagy is specific. It is known that hydrogen peroxide (H_2_O_2_) can also increase intracellular ROS levels. However, in our study, the treatment of Jurkat cells with different concentrations of H_2_O_2_ resulted in an increase in the ROS level, but did not cause accumulation of ubiquitinated proteins in the mitochondria and subsequent mitophagy (Supplementary Fig. S3a, b). In addition, we found that the widely used autophagy inhibitor CQ did not elevate ROS concentrations and the same concentration of CQ (5 μM) alone or combined with vorinostat did not induce obvious autophagy inhibitory activity in Jurkat cells. (Supplementary Fig. S4a, b). This finding may explain why QC is more effective than CQ. Based on the above results, we propose that, on the one hand, the co-treatment of QC with vorinostat promoted the increase of ROS in mitochondria, which resulted in the disruption of mitochondria and mitophagy. On the other hand, QC blocked the autophagy flux and induced the accumulation of mito-aggresomes in the cytoplasm. These actions further enhanced the mitochondria dysfunction and increased the levels of ROS, and eventually led to the death of the T-ALL cells.

This combination is less toxic to normal peripheral blood cells and no obvious side effects were observed when vorinostat and QC were used in our preclinical mice model. This is not surprising. In fact, tumor cells always have high base-level concentrations of ROS. They are more sensitive to the ROS-increasing agents. And, a great number of ROS-inducing agents have shown tumor-specific activities^38^, which is consistent with our results. More interestingly, vorinostat plus QC also increased ROS and further induced cells death in B-ALL cells (Supplementary Fig. S5a–d).

In summary, we demonstrate that the combination of vorinostat and QC is effective in inducing apoptosis of T-ALL cells in vitro and in vivo. Increased ROS levels and inhibition of mitophagy are important for the anti-T-ALL effect of the combination. Therefore, the combination of vorinostat and QC may represent a novel regimen for the treatment of T-ALL, which deserves future clinical trials.

## Materials and methods

### Cell culture, patients, and chemical reagents

Human T-ALL cell lines Jurkat and Molt-4 were purchased from the ATCC (Manassas, VA, USA) and cultured in RPMI 1640 (Gibco, Carlsbad, CA, USA) supplemented with 10% fetal bovine serum (FBS; Invitrogen). All cells were cultured and maintained at 37 °C in an incubator with 5% CO_2_. Anti-caspase-3, anti-PARP1, anti-parkin, anti-ubiquitin, anti-K63-linkage polyubiquitin, and anti-K48-linkage polyubiquitin were manufactured by Cell Signaling Technology (Danvers, MA, USA). PBMCs were isolated from the blood of healthy volunteers by Ficoll-Hypaque (Pharmacia, Piscataway, NJ, USA) density sedimentation. Vorinostat and QC were purchased from Sigma-Aldrich (St. Louis, MO, USA). Vorinostat was dissolved in 100% dimethyl sulfoxide (Solarbio, Beijing, China) to a stock concentration of 2 mM, and QC was dissolved in distilled water to a stock concentration of 50 mM, and stored at −80 °C.

### Cell viability assay

The cell viability was determined by Cell Counting Kit-8 (Dojindo, Kumamoto, Japan). Tumor cells (5 × 10^4^/well) were seeded onto 96-well plates at a total volume of 200 µL per well and treated with drugs for 24 and 48 h. Then, CCK-8 solution (10 µL) was added in each well, and the plates were incubated for another 4 h at 37 °C. The absorbance was determined at 450 nm using a Synergy H4 microplate reader (Dynex, Chantilly, VA, USA).

### Cell apoptosis assay

Quantification of apoptosis was performed by the Annexin-V Apoptosis Detection Kit (BD Pharmingen) following the manufacturer’s instructions. Annexin-V-positive and propidium iodide-negative cells were considered to be in the early apoptotic phase, whereas the cells that exerted positive staining for Annexin-V and propidium iodide were considered to be in the late apoptotic and/or necrotic stage. All data were collected, stored, and analyzed by LYSIS II software (BD Biosciences, San Diego, CA, USA).

### Cell cycle assay

Cells (2 × 10^6^) were washed twice with phosphate-buffered saline (PBS) and then fixed with 75% cold ethanol at −20 °C overnight. RNA was removed by incubation with RNase (100 mg/mL) for 30 min at 37 °C. Cell cycle distribution was analyzed by staining with propidium iodide (Sigma-Aldrich, USA; 250 mg/mL) for another 15 min at room temperature. Then, cells were analyzed by flow cytometry.

### Mitochondrial transmembrane potentials (ΔΨm) assay

Mitochondrial transmembrane potentials was determined by staining with JC-1 (5,5′,6,6′-tetrachloro-1,1′,3,3′-tetraethyl-imidacarbocyanine iodide, Beyotime, China) following the manufacture's instruction. Cells (1 × 10^6^) were treated with SAHA and/or QC for 24 or 48 h and then centrifuged and washed twice with PBS. Next, the cells were stained with JC-1 staining solution (2 μM) for 15 min at 37 °C and washed twice with PBS. The fluorescence was detected using a FACSCalibur flow cytometer (Becton Dickinson, USA). JC-1 aggregate was measured at the FL-2 channel and green fluorescent (both JC-1 monomer and/or GFP) at the FL-1 channel. The ratio of JC-1 monomers and JC-1 aggregates represented the condition of ΔΨm.

### Transmission electron microscopy

Jurkat cells (2 × 10^7^) were treated with vorinostat or/and QC for 36 h and then centrifuged and washed twice with PBS. Cells were fixed in 2% glutaraldehyde in PBS (pH 7.4) for 3 h at 4 °C, and osmicated in 1% osmium tetroxide for 2 h at 4 °C. After dehydration with a graded ethanol series, each sample was embedded in Epon812 and sectioned using a Leica EM UC6 (Leica Co., Vienna, Austria) ultramicrotome. Sections were examined by a TEM Tecnai G2 20 (FEI Co., Hillsboro, OR, USA) at 200 kV.

### Mitochondria and cytosol isolation

Isolation of mitochondria and cytosol was performed by using a Mitochondria/Cytosol Isolation Kit (Applygen Technologies, Beijing, China) according to the manufacturer’s instructions. Jurkat cells (2 × 10^7^) were washed twice with PBS and then resuspended with 1 mL of ice-cold Mito-Cyto isolation buffer. Further, the samples were placed on ice for 10 min, and the cells were homogenized on ice by Dounce Tissue Grinders (Kimble, Wheaton, USA). The homogenate was centrifuged at 1000 × *g* for 10 min at 4 °C, and the supernatants were removed to a new tube. The mitochondria were obtained by centrifugation at 15,000 × *g* for 20 min at 4 °C, whereas the cytosol was isolated by centrifugation of the remaining supernatant at 13,000 × *g* at 4 °C for 5 min using the methanol/chloroform method.

### Reactive oxygen species

ROS in Jurkat cells, which were dehydrated and showed red signals, were detected by dihydroethidium (DHE) fluorescent probe (Beyotime Biotechnology, China). The harvested cells were incubated with 10 μM DHE for 30 min at 37 °C according to the manufacturer’s instructions. The fluorescence signal was measured using a FACSCalibur flow cytometer (Becton Dickinson, USA) at an excitation wave length of 535 nm and an emission wave length of 610 nm.

### Western blot analysis

Whole cells were centrifuged and washed twice with PBS and then resuspended with cold PBS, followed by the addition of an equal volume of 2× cell lysis buffer. The protein concentration was quantified using the Bradford Protein Assay Kit (Thermo, Rockford, IL, USA). Cell lysates were separated by sodium dodecyl sulfate-polyacrylamide gel electrophoresis, and proteins were transferred to nitrocellulose filter membranes (NC) (Millipore, Billerica, MA, USA). The membranes were then incubated with the corresponding antibodies at 4 °C overnight. Next, the membranes were washed three times with TBS/T (Tris-buffered saline, 0.1% Tween-20) and then incubated with the appropriate horse radish peroxidase-conjugated secondary antibodies for 1 h at room temperature. Protein expression was detected by chemiluminescence (GE Healthcare, Piscataway, NJ, USA).

### RNA interference and transfection

Pairs of complementary oligonucleotides against ATG7 and non-target control short hairpin RNA (shRNA) (NC) were synthesized by Sangon Biotech (Shanghai, China) and annealed and ligated to the PGIPZ vector (Clontech Laboratories, Inc., Palo Alto, CA, USA). The shRNA-carrying retroviruses, which were produced in 293T cells, were used to infect Jurkat cells.

### Xenograft mouse model

Non-obese diabetes/SCID (NOD/SCID) male mice aged 4–6 weeks were used in the experiments. Jurkat cells (2 × 10^7^/0.2 mL cells in PBS) were injected subcutaneously in the right hind leg of sublethally irradiated (250 cGy) male NOD-SCID mice. Tumor growth and mouse weight were monitored every 2 days. After the tumor was palpable (tumor volume of approximately 100 mm^3^), mice were randomized into two groups, a vehicle control group and a treatment group (*n* = 4 per group). Mice were treated with vorinostat (70 mg/kg; intraperitoneally), QC (50 mg/kg; intraperitoneally), or with both agents for 12 days. Then, all mice in the groups were killed, and the tumor tissues were removed. The study was approved by the Shanghai Jiao Tong University School of Medicine Institutional Animal Care & Use Committee.

### Statistical analysis

Comparisons among groups were performed by the Student’s *t* test or Tukey–Kramer comparison test followed by analysis with GraphPad Prism software (GraphPad Software, San Diego, CA, USA). The differences were considered significant at *P* < 0.05.

## Electronic supplementary material

supplementary Figure S1

supplementary Figure S2

supplementary Figure S3

supplementary Figure S4

supplementary Figure S5

supplementary file

## References

[1] Bongiovanni D, Saccomani V, Piovan E (2017). Aberrant signaling pathways in T-cell acute lymphoblastic leukemia. Int. J. Mol. Sci..

[2] Moharram SA (2017). Efficacy of the CDK inhibitor dinaciclib in vitro and in vivo in T-cell acute lymphoblastic leukemia. Cancer Lett..

[3] Lu K (2015). The STAT3 inhibitor WP1066 synergizes with vorinostat to induce apoptosis of mantle cell lymphoma cells. Biochem. Biophys. Res. Commun..

[4] Tan J, Cang S, Ma Y, Petrillo RL, Liu D (2010). Novel histone deacetylase inhibitors in clinical trials as anticancer agents. J. Hematol. Oncol..

[5] Lemoine M, Younes A (2010). Histone deacetylase inhibitors in the treatment of lymphoma. Discov. Med..

[6] Ogura M (2014). A multicentre phase II study of vorinostat in patients with relapsed or refractory indolent B-cell non-Hodgkin lymphoma and mantle cell lymphoma. Br. J. Haematol..

[7] Apuri S, Sokol L (2016). An overview of investigational histone deacetylase inhibitors (HDACis) for the treatment of non-Hodgkin’s lymphoma. Expert Opin. Investig. Drugs.

[8] Zhang C (2015). Histone acetylation: novel target for the treatment of acute lymphoblastic leukemia. Clin. Epigenet..

[9] Xue K (2016). Vorinostat, a histone deacetylase (HDAC) inhibitor, promotes cell cycle arrest and re-sensitizes rituximab- and chemo-resistant lymphoma cells to chemotherapy agents. J. Cancer Res. Clin. Oncol..

[10] Yang B (2015). Antitumor activity of SAHA, a novel histone deacetylase inhibitor, against murine B cell lymphoma A20 cells in vitro and in vivo. Tumour Biol..

[11] Kunami N, Katsuya H, Nogami R, Ishitsuka K, Tamura K (2014). Promise of combining a Bcl-2 family inhibitor with bortezomib or SAHA for adult T-cell leukemia/lymphoma. Anticancer Res..

[12] Preet R (2016). Chk1 inhibitor synergizes quinacrine mediated apoptosis in breast cancer cells by compromising the base excision repair cascade. Biochem. Pharmacol..

[13] Wang W (2011). Quinacrine sensitizes hepatocellular carcinoma cells to TRAIL and chemotherapeutic agents. Cancer Biol. Ther..

[14] Sokal DC (2010). Quinacrine sterilization and gynecologic cancers: a case–control study in northern Vietnam. Epidemiology.

[15] Sun MG (2015). Targeting epirubicin plus quinacrine liposomes modified with DSPE-PEG2000-C(RGDfK) conjugate for eliminating invasive breast cancer. J. Biomed. Nanotechnol..

[16] de Souza PL, Castillo M, Myers CE (1997). Enhancement of paclitaxel activity against hormone-refractory prostate cancer cells in vitro and in vivo by quinacrine. Br. J. Cancer.

[17] Gallant JN (2011). Quinacrine synergizes with 5-fluorouracil and other therapies in colorectal cancer. Cancer Biol. Ther..

[18] Song P (2015). Asparaginase induces apoptosis and cytoprotective autophagy in chronic myeloid leukemia cells. Oncotarget.

[19] Changchien JJ (2015). Quinacrine induces apoptosis in human leukemia K562 cells via p38 MAPK-elicited BCL2 down-regulation and suppression of ERK/c-Jun-mediated BCL2L1 expression. Toxicol. Appl. Pharmacol..

[20] Neznanov N (2009). Anti-malaria drug blocks proteotoxic stress response: anti-cancer implications. Cell Cycle.

[21] Liang GW (2008). Enhanced therapeutic effects on the multi-drug resistant human leukemia cells in vitro and xenograft in mice using the stealthy liposomal vincristine plus quinacrine. Fund. Clin. Pharmacol..

[22] Ordureau A (2014). Quantitative proteomics reveal a feedforward mechanism for mitochondrial PARKIN translocation and ubiquitin chain synthesis. Mol. Cell...

[23] Erpapazoglou Z, Walker O, Haguenauer-Tsapis R (2014). Versatile roles of k63-linked ubiquitin chains in trafficking. Cells.

[24] Idippily ND, Gan C, Orefice P, Peterson J, Su B (2017). Synthesis of vorinostat and cholesterol conjugate to enhance the cancer cell uptake selectivity. Bioorg. Med. Chem. Lett..

[25] Chao MW (2015). The synergic effect of vincristine and vorinostat in leukemia in vitro and in vivo. J. Hematol. Oncol..

[26] Pili R (2017). Combination of the histone deacetylase inhibitor vorinostat with bevacizumab in patients with clear-cell renal cell carcinoma: a multicentre, single-arm phase I/II clinical trial. Br. J. Cancer.

[27] Torgersen ML, Engedal N, Boe SO, Hokland P, Simonsen A (2013). Targeting autophagy potentiates the apoptotic effect of histone deacetylase inhibitors in t(8;21) AML cells. Blood.

[28] Golden EB (2015). Quinoline-based antimalarial drugs: a novel class of autophagy inhibitors. Neurosurg. Focus.

[29] Eriksson A (2015). Drug screen in patient cells suggests quinacrine to be repositioned for treatment of acute myeloid leukemia. Blood Cancer J..

[30] Hasegawa H (2011). LBH589, a deacetylase inhibitor, induces apoptosis in adult T-cell leukemia/lymphoma cells via activation of a novel RAIDD-caspase-2 pathway. Leukemia.

[31] Tatsuta T (2009). Protein quality control in mitochondria. J. Biochem..

[32] Taylor EB, Rutter J (2011). Mitochondrial quality control by the ubiquitin-proteasome system. Biochem. Soc. Trans..

[33] Kim Y, Triolo M, Hood DA (2017). Impact of aging and exercise on mitochondrial quality control in skeletal muscle. Oxid. Med. Cell. Longev..

[34] Kim I, Rodriguez-Enriquez S, Lemasters JJ (2007). Selective degradation of mitochondria by mitophagy. Arch. Biochem. Biophys..

[35] Boya P (2005). Inhibition of macroautophagy triggers apoptosis. Mol. Cell. Biol..

[36] Li J (2010). Proteomic analysis revealed association of aberrant ROS signaling with suberoylanilide hydroxamic acid-induced autophagy in Jurkat T-leukemia cells. Autophagy.

[37] Aitken RJ (1997). Reactive oxygen species generation by human spermatozoa is induced by exogenous NADPH and inhibited by the flavoprotein inhibitors diphenylene iodonium and quinacrine. Mol. Reprod. Dev..

[38] Miller CP, Singh MM, Rivera-Del Valle N, Manton CA, Chandra J (2011). Therapeutic strategies to enhance the anticancer efficacy of histone deacetylase inhibitors. J. Biomed. Biotechnol..

