# Supplementary figures and images for: Vorinostat and quinacrine have synergistic effects in T-cell acute lymphoblastic leukemia through reactive oxygen species increase and mitophagy inhibition
Source: Cell Death Dis. 2018 May 22;9(6):589. doi: 10.1038/s41419-018-0679-6 (PMC5964102; doi:10.1038/s41419-018-0679-6)

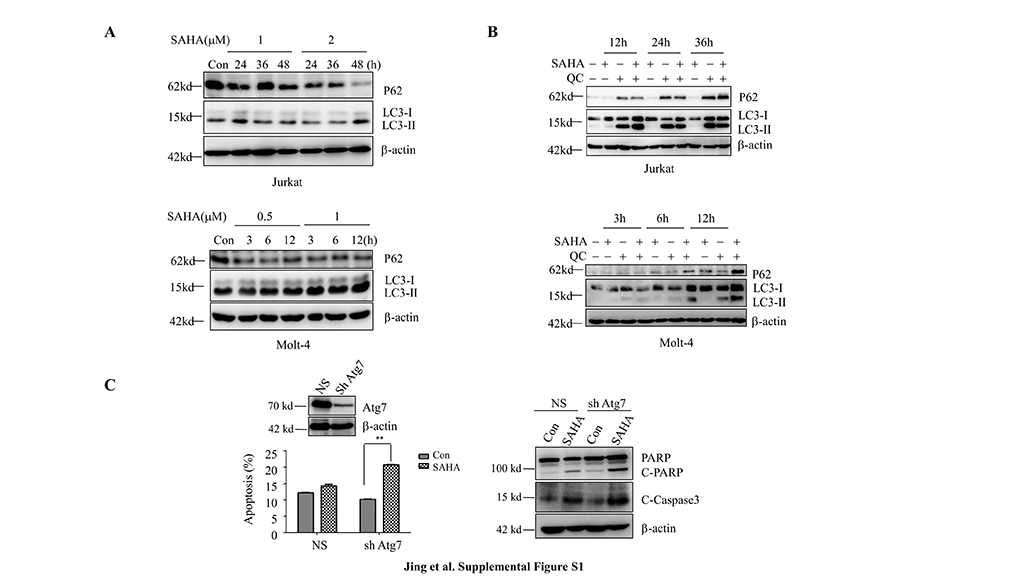

Supplement: Supplementary file 1 — supplementary Figure S1 [file 41419_2018_679_MOESM1_ESM.tif]

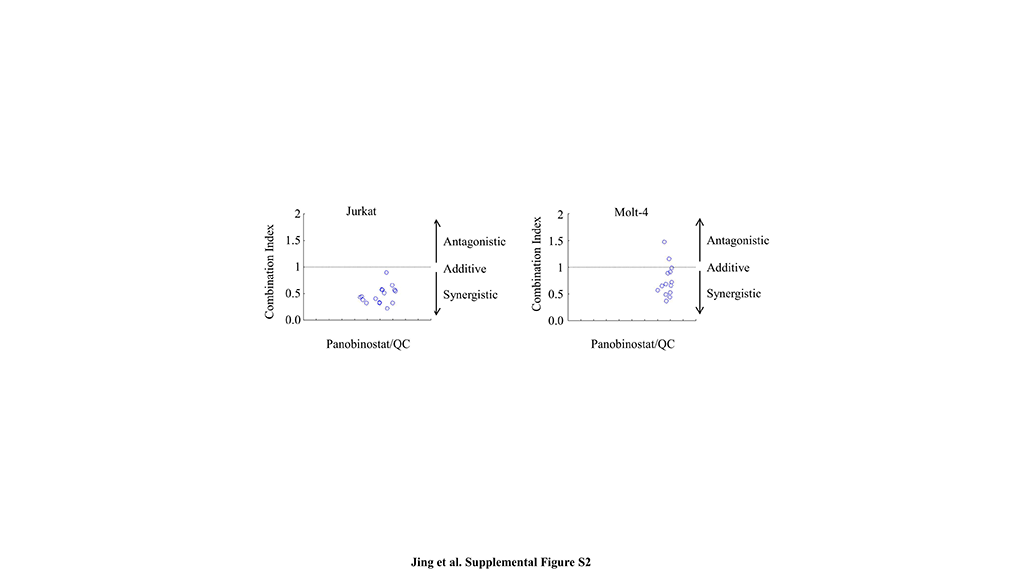

Supplement: Supplementary file 2 — supplementary Figure S2 [file 41419_2018_679_MOESM2_ESM.tif]

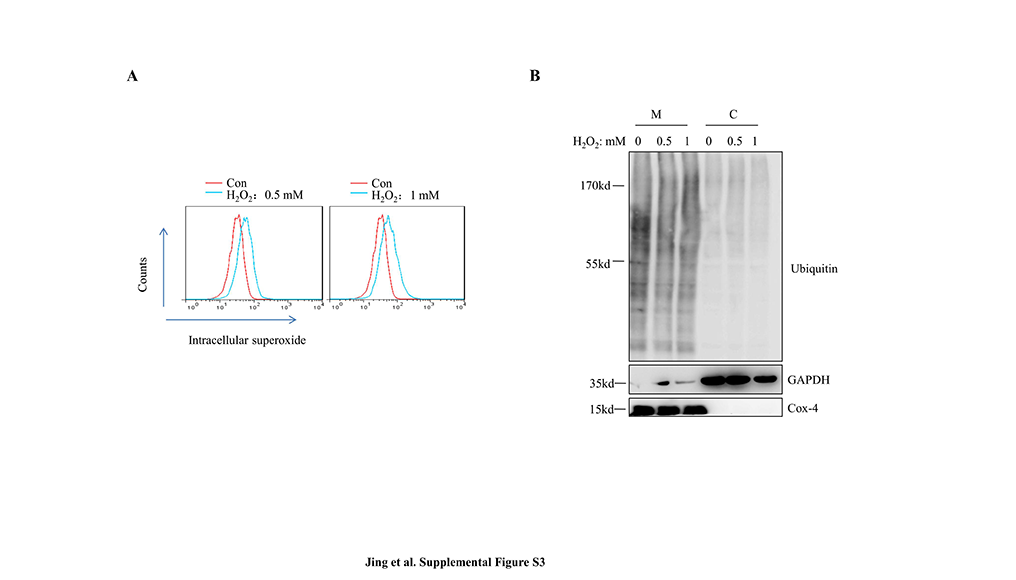

Supplement: Supplementary file 3 — supplementary Figure S3 [file 41419_2018_679_MOESM3_ESM.tif]

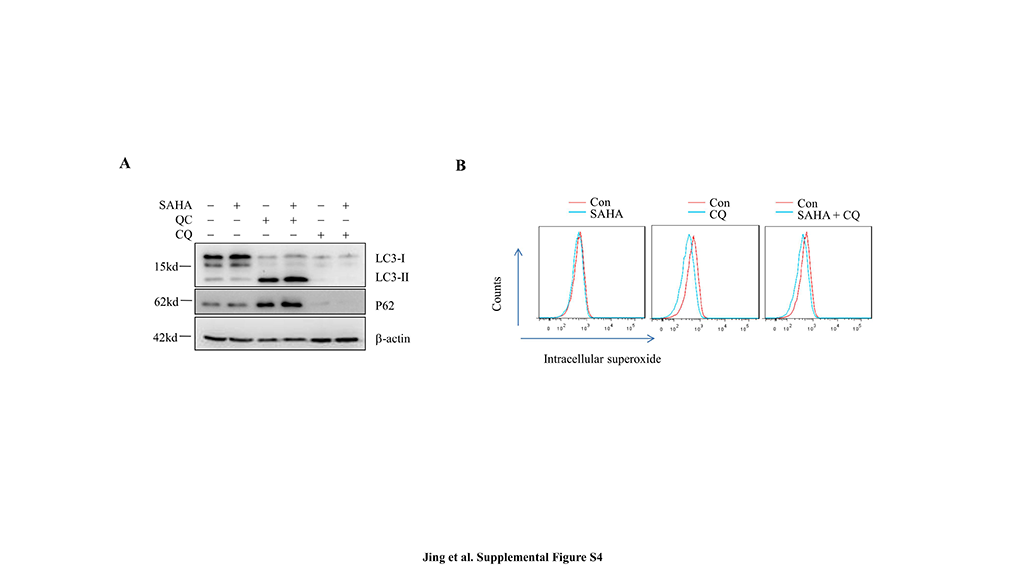

Supplement: Supplementary file 4 — supplementary Figure S4 [file 41419_2018_679_MOESM4_ESM.tif]

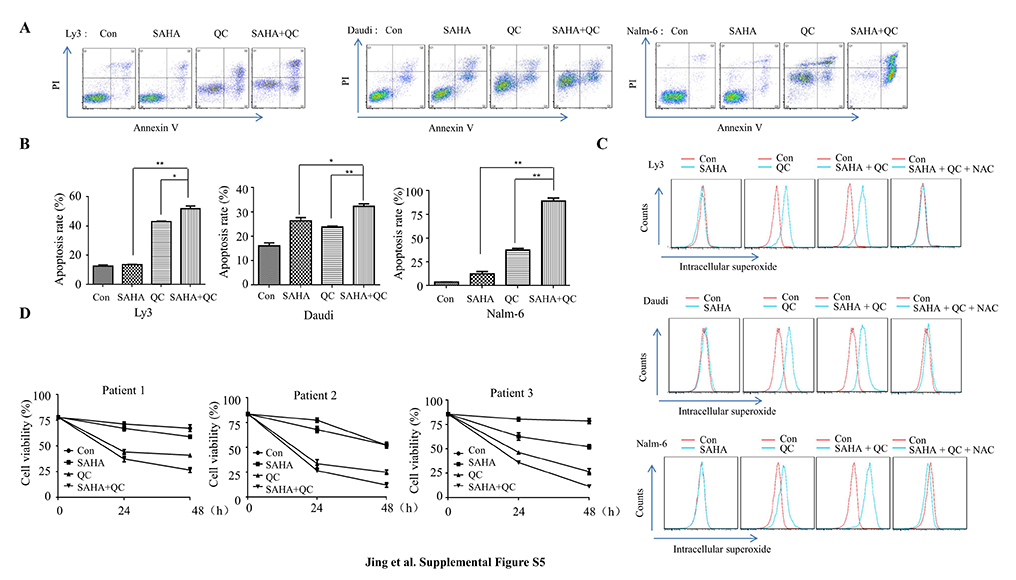

Supplement: Supplementary file 5 — supplementary Figure S5 [file 41419_2018_679_MOESM5_ESM.tif]
