# Supplementary material for: Vorinostat and quinacrine have synergistic effects in T-cell acute lymphoblastic leukemia through reactive oxygen species increase and mitophagy inhibition
Source: Cell Death Dis. 2018 May 22;9(6):589. doi: 10.1038/s41419-018-0679-6 (PMC5964102; doi:10.1038/s41419-018-0679-6)
Supplement: Supplementary file 6 — supplementary file [file 41419_2018_679_MOESM6_ESM.docx]

**Supplementary Figure Legends**

**Supplemental Figure 1****. Inhibition of autophagy enhances vorinostat-induced cell death in T-ALL cells.** **(a, b)** Jurkat and Molt-4 cells were incubated with the indicated doses of vorinostat or QC, or the combination of both for the indicated time, **(a)** P62 and LC3 were examined by Western blot; **(b)** The indicated proteins were examined by Western blot; **(c)** Jurkat cells were transfected with non-specific or Atg7 specific shRNA followed by treatment with vorinostat for 48 h. Apoptosis was determined by flow cytometry, and caspase-3 and PARP were examined by Western blot. All experiments were performed at least three times with the same results.

**Supplemental Figure 2. Panobinostat and QC have synergistic effect in T-ALL cells.** Jurkat and Molt-4 cells were treated with indicated doses of panobinostat or QC for 24 h or the combination of both, then cells viability were monitored by CCK8; CI were analyzed with the program CompuSyn.

**Supplemental Figure 3．****H_2_O_2_ does not increase the level of ubiquitinated proteins in the mitochondria of T-ALL cells. (a)** Jurkat cells were incubated with the indicated dose of H_2_O_2_ for 20 h, and DHE staining was used to analyze the ROS level in the cells; **(b)** Jurkat cells were incubated with the indicated dose of H_2_O_2_ for 24 h, and the expression levels of ubiquitinated proteins in the mitochondria and cytoplasm were examined by Western blot. All experiments were performed at least three times with the same results.

**Supplemental Figure** **4. Vorinostat combined with CQ has no effect on the level of ROS in T-ALL cells. (a)** Jurkat cells were treated with vorinostat (1 μM) and/or QC/CQ (5 μM) for 48 h and then P62 and LC3 were examined by Western blot; **(b)** Jurkat cells were treated with vorinostat (1 μM) and/or CQ (50 μM) for 24 h. DHE staining was used to determine the ROS level in the cells. All experiments were performed at least three times with the same results.

**Supplemental Figure 5．Vorinostat combined with QC has synergistic effect in B-ALL cells.** (**a, b**) Ly3, Daudi and Nalm-6 were treated with vorinostat (1 μM) and/or QC (5 μM) for 48 h and then apoptosis rate of cells was evaluated by flow cytometry analysis of Annexin V-PI dual staining; (**c**) Ly3, Daudi and Nalm-6 were treated with vorinostat (1 μM) and/or QC (5 μM) for 30 h and then ROS was analyzed by DHE staining. (**d**) PBMCs from patients with B-ALL were treated with vorinostat (1 μM) and QC (5 μM) for 24 and 48 h, then cells viability were monitored by trypan blue staining. * *p*<0.05, ** *p*<0.01, *** *p*<0.001. All experiments were performed at least three times with the same results.
